# Hemiconvulsion-Hemiplegia-Epilepsy Syndrome in Adult with Uncontrolled Seizures and Phenytoin Toxicity

**DOI:** 10.7759/cureus.7924

**Published:** 2020-05-02

**Authors:** Pal Satyajit Singh Athwal, Sandeep Aggarwal, James E Eubanks, Sukhmanii Kahlon, Parminderpal Singh

**Affiliations:** 1 Internal Medicine, Saraswathi Institute of Medical Sciences, Hapur, IND; 2 Cardiology, Geetanjali Medical College and Hospital, Udaipur, IND; 3 Physical Medicine and Rehabilitation, University of Pittsburgh Medical Center, Pittsburgh, USA; 4 Internal Medicine, Medical University of the Americas, Camps, KNA; 5 Internal Medicine, Bhagat Phool Singh Medical College, Khanpur, IND

**Keywords:** hemiplegia hemiconvulsion epilepsy syndrome, seizures, cerebral hemisphere atrophy, status epilepticus

## Abstract

Hemiconvulsion-hemiplegia-epilepsy (HHE) syndrome is a rare condition, characterized by sudden onset of unilateral seizures leading to cerebral hemisphere atrophy and hemiplegia which might persist for lifetime. It is believed to be outcome of prolonged or unmanaged status epilepticus in pediatric age group. HHE is diagnosed during childhood but we report an undiagnosed case of 30-year-old male who was dealing with uncontrolled seizure, phenytoin toxicity and hemiparesis. He was diagnosed with HHE based on characteristic imaging findings leading to complete alteration of management and opened wide array of surgical options to manage this debilitating condition.

## Introduction

Sequel to uncontrolled status epilepticus, hemiconvulsion-hemiplegia-epilepsy (HHE) syndrome was first described by Dr. Henry Gastaut in 1964 [1]. HHE syndrome is a sudden onset of unilateral convulsions seizure which progresses to cerebral hemisphere atrophy and varying degree of hemiplegia and epilepsy which can persist life long. It begins with hemispherical cerebral edema later on leading to atrophy. It has been reported before as sequel to status epilepticus and few cases following partial seizures or secondary generalized tonic clonic seizures [2]. Dr. Gastaut divided this condition into two types. Type 1 which is a sequel to encephalitis, meningitis or vascular lesions and type 2 is idiopathic. Incidence has been decreased dramatically due to aggressive treatment of seizures. Association with hypercoagulability state such as proteins deficiency, Factor V laden mutation and L2 hydroxyglutamic aciduria is also reported in some cases [3-4]. It is a condition which presents in childhood and diagnosed early. In this case, the patient remained undiagnosed for years and was treated as epilepsy.

## Case presentation

**Figure 1:**
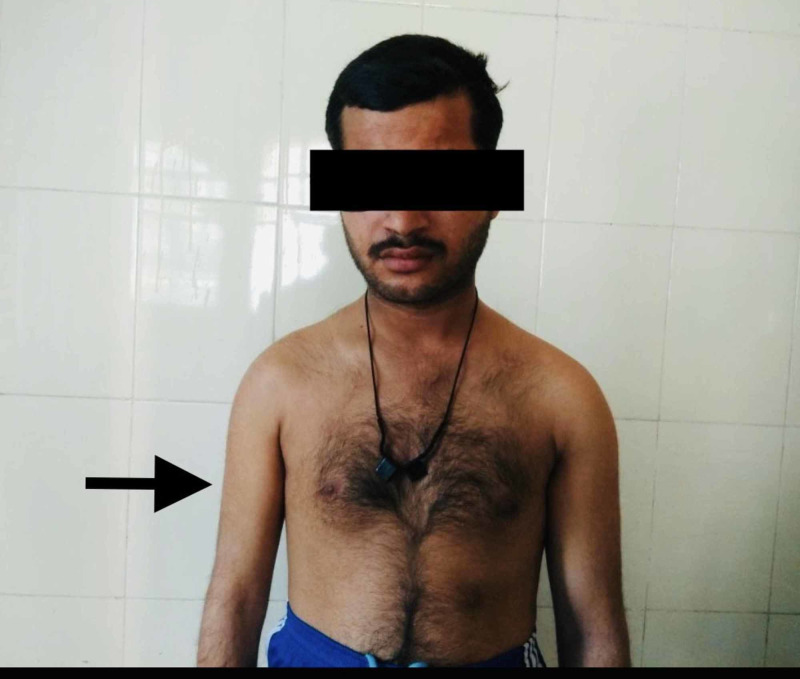
Right-sided muscular atrophy.

**Figure 2:**
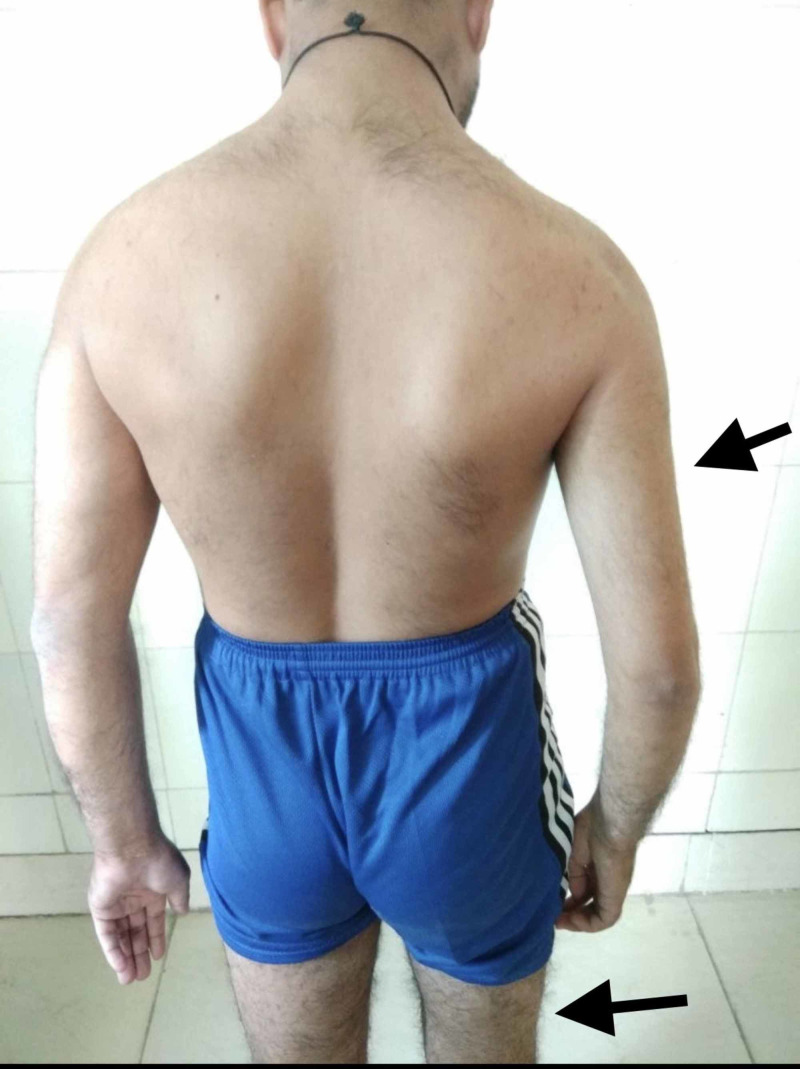
Muscular atrophy over right side compared to the left side.

A 30-year-old male presented with complaint of uncontrolled right-sided hemiconvulsions and right-side weakness since childhood. Hemiconvulsion lasts 10-15 min with variable frequency per day. He was treated with different antiepileptic medication which eventually failed and the patient was referred to Sarawathi Institute of Medical Sciences. The patient also complained of difficulty balancing, changes over gums and nausea which started few days ago. The patient recently changed the dose of phenytoin which was prescribed by general practitioner. Examination revealed gingival hyperplasia, normal cardiovascular and respiratory examination. On central nervous system examination there was right-sided hemiparesis along with hypertonia, hyperreflexia and extensor plantar reflex. Right-sided muscular atrophy was clearly observed as shown in Figure 1 and Figure 2.

**Figure 3:**
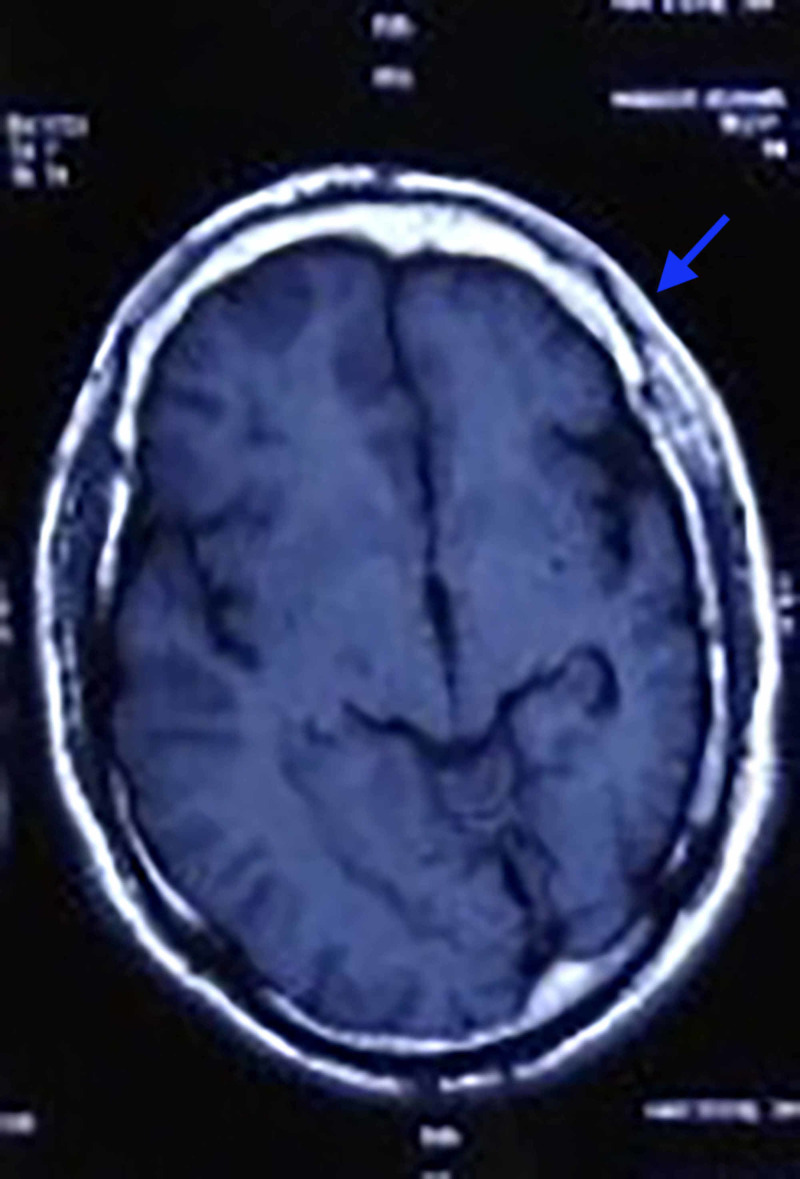
Left-sided cerebral atrophy as seen on magnetic resonance imaging.

Complete blood count, basic metabolic panel, liver function and kidney function were under normal limits. Coagulation studies including prothrombin time, bleeding time and partial thromboplastin time were normal. Serum phenytoin level was above therapeutic levels. On MRI, a general atrophy of the entire left cerebral hemisphere with dilation of ventricles was noticed (Figure 3).

Based on the history of the patient along with examination findings and imaging diagnosis, hemiconvulsion-hemiplegia syndrome was established. The patient was started on carbamazepine and phenobarbitone along with physiotherapy of involved limbs. The patient was referred to the neurosurgery department later on for surgical management if possible. Phenytoin was discontinued without any sign of dysrhythmia on ECG.

## Discussion

Hemiconvulsion-hemiplegia syndrome is characterized by convulsive seizures involving one side of the body followed by hemiplegia and epilepsy. It is usually diagnosed during childhood but this case remained undiagnosed for years. It is classified into type 1 which is followed by meningitis, encephalitis or some vascular lesions and type 2 which is idiopathic post status epilepticus [1]. No evidence of vascular lesion was seen on imaging. Normal coagulation studies ruled out any hypercoagulability state as association with this condition is reported by some authors [3-4]. Human herpesvirus 6 infection, CACNA1A gene mutation and coalition disorders like protein C and S deficiency might be one of the etiological factors. Cognitive functions and linguistic skills remained normal in this case thus ruling out Rasmussen's encephalitis (Table 1) [5].

**Table 1:** Differential diagnosis.

Differential Diagnosis:
Rasmussen’s Encephalitis
Stroke
Infections-Meningitis, Encephalitis
Neoplasm
Congenital brain malformations

The pathophysiologic mechanism includes prolonged seizures leading to excitotoxic cell injury or damage to blood-brain barrier permeability leading to cerebral edema. Predisposing factors can be genetic or focal epileptogenic lesions [6]. Disease usually begins with febrile illness and might have episodes of febrile or afebrile seizures (Table 2) [7].

**Table 2:** Symptoms and signs of hemiconvulsion hemiplegia syndrome.

Symptoms	Signs
Recurrent Hemiconvulsions	Hyperreflexia
Febrile illness	Hypertonia
Weakness involving half of body	Extensor plantar response
	Muscular atrophy of the side involved

Diagnosis can be established based on typical history and imaging. Status epilepticus or febrile seizures in children should be managed aggressively. Imaging should be performed in the acute stage because of cerebral edema-induced herniation risk. Management of delayed epilepsy involves the combination of carbamazepine and phenytoin [8]. This combination helped the patient to control seizures. Surgical interventions like hemispherectomy or corpus callosotomy can be helpful in refractory cases [9]. NMDA antagonist should be researched in the acute phase and cerebral edema should be managed as early as possible.

## Conclusions

Hemiconvulsion-hemiplegia syndrome is a rare but serious condition which can lead to life-long impact on the quality of life. It is important to prevent this condition by managing seizures appropriately and awareness about this condition is also important. This patient remained undiagnosed for years suffering from uncontrolled seizures and hemiparesis. Through this case report, we want to discuss the diagnosis and management of this rare condition.
